# Analysis of acquired mutations in transgenes arising in Ba/F3 transformation assays: findings and recommendations

**DOI:** 10.18632/oncotarget.15392

**Published:** 2017-02-16

**Authors:** Kevin Watanabe-Smith, Jamila Godil, Anupriya Agarwal, Cristina Tognon, Brian Druker

**Affiliations:** ^1^ Cancer Biology Program, Oregon Health & Science University, Knight Cancer Institute, Portland, OR, USA; ^2^ Honors College, College of Science, Oregon State University, Corvallis, OR, USA; ^3^ Division of Hematology and Medical Oncology, Oregon Health & Science University, Knight Cancer Institute, Portland, OR, USA; ^4^ Molecular and Medical Genetics, Oregon Health & Science University, Portland, OR, USA; ^5^ Oregon Health & Science University, Knight Cancer Institute, Portland, OR, USA; ^6^ Howard Hughes Medical Institute, Portland, OR, USA

**Keywords:** reproducibility in research, Ba/F3 transformation assay, functional validation, leukemia, oncogenes

## Abstract

The identification and functional validation of potentially oncogenic mutations in leukemia is an essential step toward a future of personalized targeted therapy. To assess the oncogenic capacity of individual mutations, reliable and scalable *in vitro* experimental approaches are required. Since 1988, researchers have used the IL-3 dependent Ba/F3 transformation assay to validate the oncogenic potential of mutations to drive factor-independent growth. Here we report a previously unrecognized phenomenon whereby Ba/F3 cells, engineered to express weakly transforming mutations, present with additional acquired mutations in the expressed transgene following factor withdrawal. Using four mutations with known transformative capacity in three cytokine receptors (CSF2RB, CSF3R and IL7R), we demonstrate that the mutated receptors are highly susceptible to acquiring additional mutations. These acquired mutations of unknown functional significance are selected by factor withdrawal but appear to exist prior to the removal of growth factor. This anomaly has the potential to confound efforts to both validate and characterize oncogenic mutations in leukemia, particularly when it is not standard practice to sequence validate cDNAs from transformed Ba/F3 lines. We present specific recommendations to detect and mitigate this phenomenon in future research using Ba/F3 transformation assays, along with methods to make the Ba/F3 assay more quantitative.

## INTRODUCTION

In 1988, George Daley and David Baltimore established that BCR-ABL—the gene fusion product found in almost every case of chronic myelogenous leukemia (CML) [1]—was oncogenic using the murine Ba/F3 cell line [2]. This cell line, derived from pro-B bone marrow cells, required interleukin 3 (IL-3) for normal proliferation and survival [3]. Stable expression of BCR-ABL in Ba/F3 cells blocked apoptosis and promoted proliferation, resulting in IL-3 independent growth [2] and, as a result, a new transformation assay was created. The Ba/F3 system was a superior model for hematopoietic malignancies in contrast to earlier NIH 3T3 growth-foci formation assays, where the BCR-ABL fusion was insufficient to transform fibroblasts [4].

In a typical Ba/F3 transformation assay, Ba/F3 cells are infected by a retrovirus or electroporated with a plasmid, driving expression of a gene of interest. Cells expressing the transgene are selected for by FACS or antibiotic treatment based on additional markers within the vectors, and then washed to remove IL-3 from the culture medium to initiate a cytokine-independent growth assay. Growth is monitored for two or three weeks. If cells continue to proliferate it is concluded that the transgene is transformative and an active oncogene. The resulting transformed Ba/F3 cells are frequently used for pathway analysis to characterize mechanisms of oncogenic signaling or to test the effects of selective targeted inhibitors on cell growth and survival [5]. A meta-analysis of all articles, published between 2014 and 2016, using the Ba/F3 system indicates that the cells are not sequenced following oncogenic transformation to detect the presence of additional mutations in the expressed transgene [6–29] (Table 1, Supplemental Table 1).

**Table 1:** Frequency of sequence validation in Ba/F3 transformation studies (2014-2016)

Characteristics		PubMed Articles (%)	
Publication Year			
	2014	8	(33)
	2015	9	(38)
	2016	7	(29)
Search Term			
	Ba/F3	23	(96)
	Baf3	1	(4)
Method of transduction			
	Retrovirus	12	(50)
	Lentivirus	2	(8)
	Electroporation	6	(25)
	Unclear	4	(17)
Sequencing outgrown Ba/F3 lines			
	Transgene sequence confirmed	0	(0)
	Transgene not sequenced	24	(100)

Source: PubMed articles matching “Ba/F3” or “Baf3”, published 2014-2016, and using Ba/F3 cells for the purpose to establish transformative potential of genetic products. Data current as of 10-26-16.

While testing a transforming CSF2RB mutation in a previous study [30], we noted that CSF2RB R461C-expressing cells demonstrated two reliable phenotypes: IL-3 independent growth and acquired mutations in the CSF2RB transgene following IL-3 withdrawal. Interestingly, when the CSF2RB wildtype-expressing cells spontaneously transformed it was only through the acquisition of a previously characterized transforming mutation (V449E) [31]. Conversely, R461C-expressing cells acquired a series of divergent mutations that were found throughout the receptor.

These observations led us to expand our investigation to include additional genes with well-established activating mutations in order to determine the prevalence and rate of acquired mutations that occur in this system. We found that acquired mutations are not unique to CSF2RB R461C but are similarly present in CSF3R variants. These acquired mutations are more common in weakly-transforming constructs. Withdrawal of IL-3 serves as a selective pressure to enrich for acquired mutations, but our data suggest these mutations exist prior to factor withdrawal. In contrast to earlier findings [32], we find no evidence that the time spent in culture between viral infection and factor withdrawal influences transformation rate. We recommend that future studies using the Ba/F3 transformation assay verify the complete sequence of the ectopically expressed transgene prior to pathway characterization and publication of results. These recommendations are particularly critical for weakly or slowly transforming mutations, where acquired mutations appear to be most prevalent.

## RESULTS

### Experimental design

Three cytokine receptors harboring four unique oncogenic mutations along with wild type control receptors were selected for this study based upon their well-established transformation capability (Table 2). CSF2RB R461C is a germline mutation found in a T-ALL patient and shown to activate ligand-independent signaling *in vitro* [30]. CSF3R T618I is a prominent mutation in CNLs and aCMLs and leads to ligand-independent activation [33]. CSF3R truncation mutations (including W791X) have also been reported in both CNL and aCML [33], resulting in surface receptor accumulation through altered endocytosis and degradation [34, 35]. IL7R 243InsPPCL was described in a B-ALL patient [36] and is one of a group of mutations in pediatric ALLs that activate IL7R by introducing unpaired cysteine residues in the membrane-proximal region of the receptor that cause constitutive dimerization [37]. Expression of BCR-ABL fusion or empty vector served as positive and negative controls, respectively, for transformation of Ba/F3 cells in our assays.

**Table 2:** Constructs used in this study

Gene	Mutation
CSF2RB	WT
CSF2RB	R461C
CSF3R	WT
CSF3R	T618I
CSF3R	W791X
IL7R	WT
IL7R	243InsPPCL
Empty Vector	n/a
BCR-ABL	BCR-ABL (p210)

**Figure 1:**
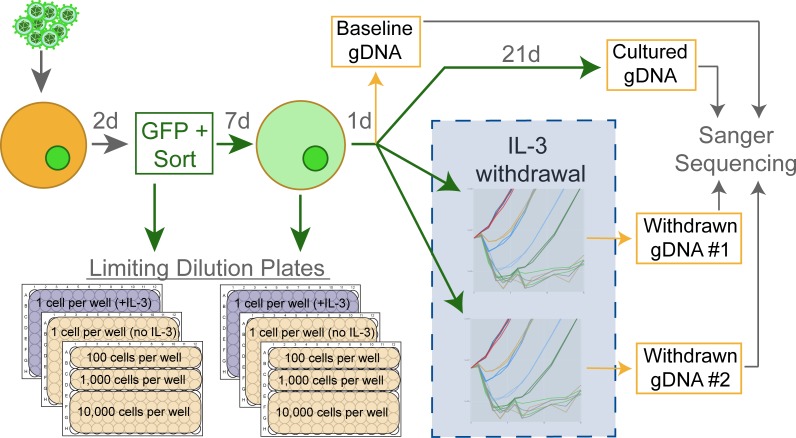
Experimental design schematic Ba/F3 cells are infected using freshly harvested retrovirus which drives the expression of GFP and the transgene of interest. Infected cells are selected for GFP-expression on day 2, and the first set of limiting dilution plates are created. On day 9 a second set of limiting dilution plates are started, and on day 10 the cells are triple-washed then monitored for IL-3 independent growth over 21 days in two technically replicate flasks. Genomic DNA (gDNA) is harvested from cell lines in IL-3 on day 10 (baseline) and day 31 (cultured). As cells proliferate in the absence of IL-3, gDNA is harvested to detect acquired mutations. This workflow was repeated for every construct to create a minimum of three biologically replicate lines.

**Figure 2:**
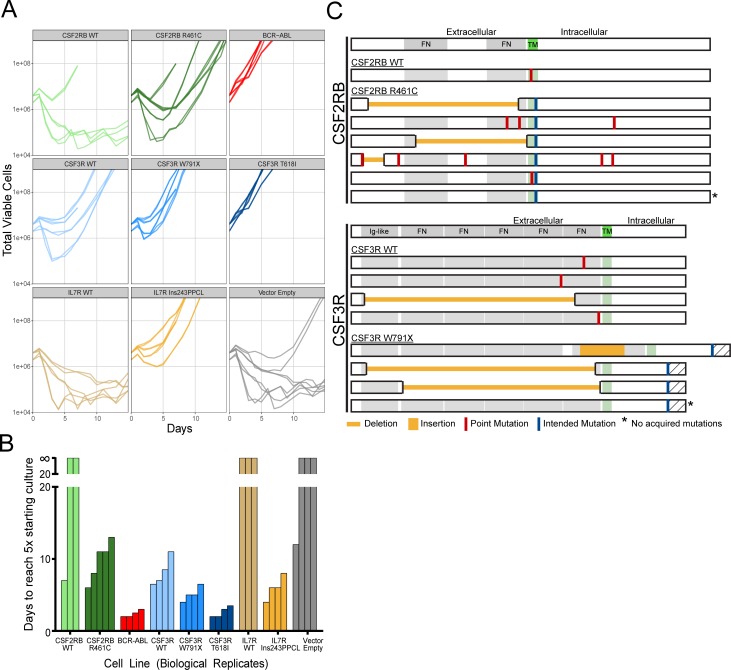
Compiled data from all IL-3 withdrawal experiments **A**. Outgrowth curves for every replicate in this study, separated by transgene and mutation. The outgrown vector empty line was sequenced and found to be contaminated with IL7R Ins243PPCL. **B**. Time to outgrowth can be summarized as the number of days to reach a viable cell count 5-times the number of cells initially seeded in each flask at the start of the experiment. Lines that did not grow within the 21-day period are shown above the break. **C**. Mutations detected in outgrown lines after transformation to factor-independent growth. Every transformed line for CSF2RB WT, R461C and CSF3R WT, W791X are shown, including lines that did not acquire additional mutations (*).

The schematic diagram in Figure 1 describes the protocol used to create and collect samples for subsequent sequence verification. Retrovirally infected Ba/F3 cells were sorted by GFP-positive FACS 48-hours post-infection and a series of limiting dilution plates were created. On day 9, a second series of limiting dilution plates were created. On day 10 each flask was triple-washed and split into replicate flasks to monitor IL-3 independent growth. Cells undergoing IL-3 withdrawal were monitored using a Guava ViaCount for 21 days. While variability was evident between biological replicates, technical replicate flasks from the same infected cell line exhibited near identical outgrowth curves (Figure 2A). Time to factor-independent growth is represented by the number of days to reach 500% of viable cells in the initial culture (Figure 2B).

Genomic DNA (gDNA) was immediately harvested from every outgrown line once factor-independent proliferation was evident. Genomic DNA was harvested from cell lines cultured in IL-3 containing media at the point of factor withdrawal (day 10) and also at day 31, which represents the complete length of the transformation assay. Transgenes were PCR amplified from gDNA using vector-specific primers, Sanger sequencing was performed, and all mutations from every cell line were comprehensively catalogued.

### Acquired mutations detected in transformed lines

Sanger sequencing of these cell lines revealed acquired mutations in the one CSF2RB WT line that transformed (1 of 3) and every CSF3R WT line (4 of 4), all of which transformed (Figure 2C, Supplemental Table 2). The majority of CSF2RB R461C lines (4 of 5) and CSF3R W791X lines (3 of 4) also presented with a variety of additional acquired mutations (Figure 2C). The CSF3R T618I lines were the most rapidly transforming and did not contain acquired mutations, nor did the slightly slower-growing IL7R 243InsPPCL lines. The empty vector (negative control) transformed once and sequencing revealed a contamination with IL7R Ins243PPCL in both replicate flasks, demonstrating the ability to detect cross-contamination in our experimental design. In all but one case (11 of 12), replicate withdrawal flasks exhibited identical acquired mutations upon sequencing (Supplemental Table 2), indicating the acquired mutations were likely present prior to IL-3 withdrawal. To rule out the possibility that acquired mutations are merely a function of time in culture, we sequenced the transgene of cells on the day of withdrawal (day 10, baseline) and again following 21 days in culture with IL-3 (day 31, cultured). Sequencing revealed mutations in only 1 of 15 cultured lines (Supplemental Table 2), indicating that the acquired mutations do not provide a competitive, proliferative advantage while growing in IL-3-supplemented media.

### CSF2RB R461C does not drive genomic instability

The high rate of acquired mutations in CSF2RB R461C, both reported here and observed in our prior experiments, led us to question if the variant caused widespread mutagenesis or genomic instability. To address this, we performed a 6-thioguanine (6-TG) survival assay following retroviral infection. In this assay, only cells that possess a higher capability to induce mutations through genomic instability or other mechanisms will be capable of mutationally inactivating HPRT to survive 6-TG treatment. We observed no increase in survival for CSF2RB R461C cells compared to controls (ENU-treated and no infection) or any other transformed cell line (Supplemental Figure 1), indicating that the CSF2RB R461C mutation is not causing genetic instability.

### Transformation rate determined by limiting dilution analysis

In contrast to bulk Ba/F3 transformation assays, a limiting dilution assay has the ability to pinpoint the proportion of infected cells capable of surviving IL-3 withdrawal. Using this more quantitative assay we investigated whether acquired mutations are more common in lines with weaker rates of transformation. Limiting dilution plates were visually scored for growth after three weeks. Plating efficiency was calculated from plates diluted to 1 cell per well in media containing IL-3. The remaining plates were used to calculate the ratio of transforming cells (displayed as transformation rate X, where 1 in X cells are capable of factor-independent growth) with 95% confidence intervals using Extreme Limiting Dilution Analysis (ELDA) as previously described [38].

**Figure 3:**
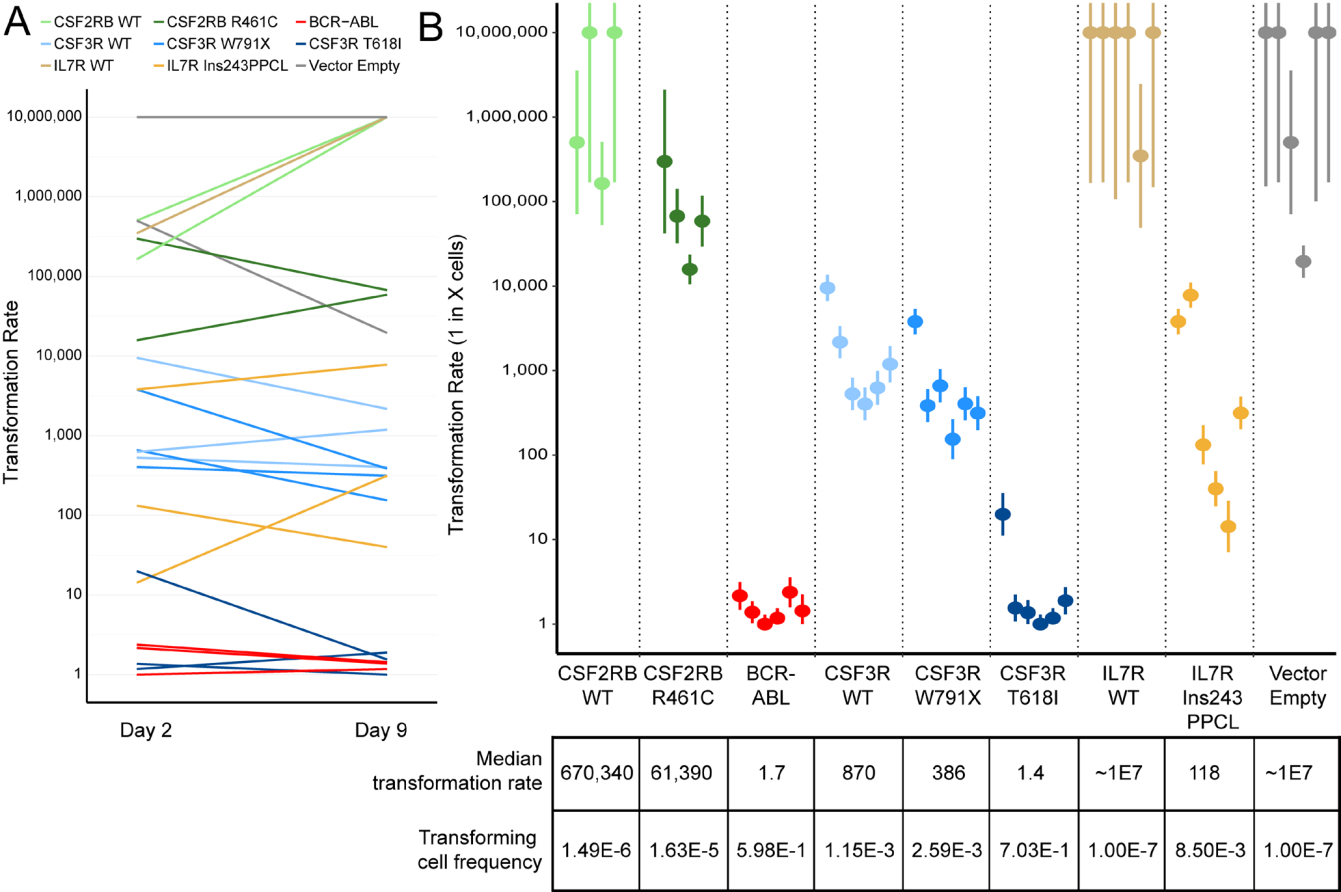
Ba/F3 transformation rates vary by transgene but not by time in culture **A**. Transformation rates calculated for each biologically replicate cell line are shown for plates started 2 days and 9 days after retroviral infection. The transformation rate is expressed as 1 in X cells capable of transforming to IL-3 independent growth, therefore a higher transformation rate indicates a weakly transforming cell line. No consistent trend is observed between days post-infection and transformation rate. **B**. Transformation rate and 95% confidence intervals for every replicate. The median rates across biologically replicate samples are shown below, along with the frequency of transforming cells (inverse of transformation rate). Lines that exhibited no observable transformation are shown with a rate of 1.0×10^7^.

A previous study investigating another *in vitro* transforming CSF2RB mutation found that the transformation rate of infected cells increased with the time in culture after viral infection and before factor withdrawal [32]. In our study, we observed no consistent effect of culture time on transformation rate with any of the mutations tested (Figure 3A). Transformation rate estimates were calculated and these results demonstrated reliable differences between individual mutations (Figure 3B). These data are also summarized by the median transformation rate, which is calculated after combining the transformation rates of the technical replicates for each mutation. Transformation rate correlated with time to outgrowth, but also served as a more quantitative measure to assess the relative functional impact of a given mutation. These data highlight the quantitative difference between strongly transforming mutations (CSF3R T618I, BCR-ABL) and weakly transforming mutations (IL7R 243InsPPCL, CSF3R W791X, CSF2RB R461C).

### Acquired mutations are exclusively observed in weakly transforming oncogenes

**Figure 4:**
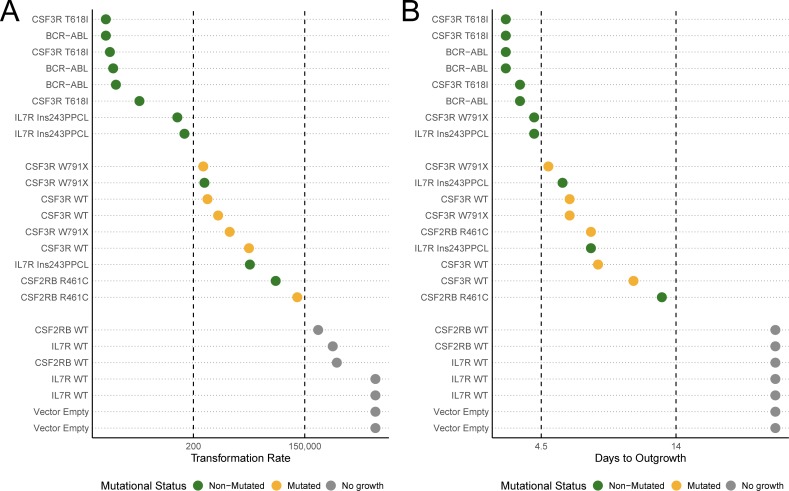
Acquired mutations occur in weak, but not strong, transforming transgenes **A**. Cell lines are ordered by transformation rate and colored based on mutational status as observed from sanger sequencing of bulk outgrowth assays of the same lines. **B**. Cell lines are ordered by days to outgrowth (time to reach a 5-times increase in viable cells over the starting cell number) and colored based on mutational status. BCR-ABL was not fully sequenced due to length and structural complexity.

Mutations that possess a weaker ability to transform cells (less than 1 in every 200 cells, Figure 4A) or a slower time to outgrowth (5 days or longer to reach a 5x increase over the initial cell number, Figure 4B) account for every case of acquired mutations in this study. While a weakly transforming mutation does not always indicate the presence of acquired mutations, our data indicate that strongly transforming mutations do not present with additional mutations.

## DISCUSSION

In a previous study, we observed that exogenous expression of the CSF2RB R461C transgene frequently presented with additional acquired mutations in the gene following selection of Ba/F3 cells to IL-3-independent cell growth. As a result of this observation, we expanded our investigation to include other transforming mutations and determine if: 1) acquired mutations also arose in other transgenes, 2) whether the mutations were present prior to IL-3 withdrawal, 3) if they were enriched in weakly transforming oncogenes, and 4) if the time in culture following infection impacted the transformation rate.

Our study indicates that weakly transforming mutations in both CSF2RB and CSF3R frequently present with acquired mutations. While this could be indicative of the large number of potentially activating mutations in each gene [31, 33, 39], it is striking that CSF3R T618I does not present with acquired mutations and expression of CSF2RB WT only infrequently results in factor-independent growth. Instead, it appears that the frequencies of these mutations are dependent on the transformation rate intrinsic to each specific oncogene.

Our data indicate that the majority of these mutations likely exist prior to factor withdrawal—replicate withdrawal flasks present with identical mutations in almost every case—but only expand to levels detectable by Sanger sequencing in the absence of IL-3. Selection of factor independent Ba/F3 cells in the absence of IL-3 has been previously proposed, possibly as the result of enrichment for cells with stronger activation of downstream signaling [40]. Retroviral infection and reverse transcription is an inherently mutagenic process [41], making it an inevitability that an infected cell line has a library of clonal mutations in the infected transgene. The number of these clonal mutations would by increased by the use of high viral titers. Based on our data, we calculate that in many cases the strength of the intended mutation (CSF3R T618I) gives up to 70% of clones (1 in every 1.4 cells) the capacity to survive factor withdrawal (Figure 3B). In these cases acquired mutations would never expand beyond the mutation detection threshold of Sanger sequencing, which is around 5-10% [42]. In other cases, the acquired mutations can be functionally active, turning a non-transforming gene into a putative oncogene, as observed with the V449E mutation found in the CSF2RB wildtype receptor (Supplemental Table 2). Then in a third, more complex case there are oncogenic constructs that are functionally active—both CSF2RB R461C and CSF3R W791X have higher transformation rates than their WT counterparts—but regularly present with acquired mutations following transformation. In this third case, the acquired mutations could be passenger mutations resulting from the rare clone capable of factor-independent growth (as rare as 1 in 60,000 cells in CSF2RB R461C; Figure 3B), or they could be functional mutations that drive factor independent growth, resulting in clonal expansion detectable by Sanger sequencing. Distinguishing between the passenger mutations and the functionally active acquired mutations would require laborious empirical investigation, detracting from the scalability of this model system and thus making it an impractical solution. We have not attempted to determine the functionality of observed acquired mutations, though specific extracellular deletions have been previously shown to result in receptor activation of CSF2RB [32, 43].

Another potential complication in transformation assays is the acquisition of mutations outside of the intended transgene. Other researchers have detailed the potential for IL9Rα mutant Ba/F3 cells to gradually reach growth factor independence through the acquisition of recurrent JAK1 mutations [44]. Additionally, the use of retroviruses can result in insertional mutagenesis, possibly disrupting tumor suppressor genes resulting in factor-independent growth. These artifacts would not be detected in our experimental design, but underscore the importance of screening biological replicates to control for false-positives. Non-transgenic mutations could also be controlled for by inducible transgene expression, but this would not solve the problem of acquired mutations within the transgene.

It is not clear what constitutes a weakly transforming mutation which drives oncogenic growth in only a subset of infected cells. The Ba/F3 model has traditionally been very effective for screening mutations in growth factor receptors [33, 37, 45–47], but in these cases of weakly transforming mutations an alternative cell line could yield more robust results. It is possible that weak mutations are barely capable of transforming cells to become factor independent, possibly requiring a high level of expression, and the majority of cells fail to reach that state. It is also possible that some mutations need a second, cooperating mutation as proposed by earlier studies [32].

Stocking and colleagues have previously described a truncated form of CSF2RB, Δβ_c_, that was capable of transforming FDC-P1 factor-dependent cells [43, 48]. In a follow-up study [32], they described two striking observations. First, 7 of 8 Δβ_c_ transformed lines could have Δβ_c_ removed by Cre-Lox excision and maintain factor independent growth. Second, Δβ_c_ lines showed no increase in transformation (by limiting dilution analysis) 24 hours after infection, but a substantial increase 10 days after infection (from 1.0×10^-8^ to 6.8×10^-5^). This led the authors to conclude that Δβ_c_ was either mutagenic—capable of creating oncogenic lesions to sustain growth—or synergistic with a spectrum of potential secondary mutations that could replace dependence on the original transgene [32]. While we do not see an effect of culture time on transformation rate in our study, and CSF2RB R461C does not appear cause genomic instability by 6-TG selection, it is possible that some mutations have the ability to synergize with a larger library of secondary cooperating mutations that might be selected in a transformation assay.

In any case, determining the significance of acquired mutations would require time-consuming independent validation. Yet the presence of these unintended mutations has the potential to confound our ability to functionally characterize potentially transforming mutations. Researchers are increasingly using transformed Ba/F3 cells to screen oncogenes for drug sensitivity [5]. In these experiments, an acquired mutation could dramatically alter results, stressing the importance of understanding this phenomenon. At this time, we cannot propose a method that eliminates the appearance of acquired mutations. Our previous study mitigated this effect by using electroporation instead of retroviral infection, avoiding the potential for reverse-transcription induced mutagenesis. However, some of these electroporated lines still presented with acquired mutations, and there is insufficient evidence to determine if this approach reduced their frequency.

We recommend that every outgrown Ba/F3 line should be sequenced to validate the sequence of the full transgene, ensuring reproducible results and reducing the risks of characterizing artificial oncogenes. Even in lines that repeatedly transform, biological replicates are prone to frequent acquired mutations. This sequence validation is particularly important for all mutations that are weakly transforming. We present our criteria for classifying a weakly transforming mutation by limiting dilution analysis (fewer than 1 in every 200 cells sustaining factor-independent growth), or time to outgrowth (lines that take more than 4 days post-factor withdrawal to reach 5x the number of viable cells in the starting culture). However, these measures could vary by protocol or laboratory and we cannot conclude that strongly transforming genes never acquire mutations. Transformation rate as determined by limiting dilution analysis would also represent a valued addition to the Ba/F3 assay. Widespread adoption of this measure in future studies would allow detailed investigation into correlations between transformation rate and clinical relevance of weakly transforming mutations.

The Ba/F3 transformation assay remains an invaluable tool for the functional validation of activating mutations found in primary leukemias. We report a previously unrecognized phenomenon where weak transforming mutations are susceptible to acquiring additional mutations within the transgene of interest. These acquired mutations could jeopardize attempts to characterize the signaling mechanisms and drug sensitivities of leukemic oncogenes. We propose an addition to the standard Ba/F3 protocol and suggest the sequencing of the full transgene in outgrown cells to detect confounding mutations and improve the reproducibility of future studies. Additional research should be directed toward methods that reduce the incidence of acquired mutations in these critical assays. Special attention should be paid to whether the use of retroviruses increases this phenomenon, as this method is the most frequently used in current studies (Table 1). A larger study covering additional leukemic oncogenes would indicate if acquired mutations occur in non-cytokine receptors, and would help define the range of studies that could be affected by this phenomenon.

## MATERIALS AND METHODS

### Cell culture

Ba/F3 cells were obtained from ATCC and grown in RPMI 1640 medium with 10% FBS, L-glutamine, fungizone, penicillin-streptomycin, and 15% WEHI-conditioned medium (a source of IL-3). Frozen vials of cells, previously in culture less than 30 cumulative days, were freshly thawed for each experiment.

### Ba/F3 transformation assay

Gene constructs were cloned as previously described [33] into a MSCV-IRES-GFP retroviral vector [49] and confirmed by Sanger sequencing. Retrovirus was created by transfecting plasmids into 293T/17 cells along with the pIK6.1MCV.ecopac.UTD helper plasmid. Virus was harvested and used to infect Ba/F3 cells in two rounds of retroviral spin inoculation. Infected Ba/F3 cells were 42% GFP-positive on average, as determined by FACS. Percent of GFP-positive cells following infection varied primarily by replicate set and minimally by transgene (average per non-control transgene ranged from 38-50%). The number of vector copies integrated into each line is unknown, but Supplemental Table 2 indicates some lines possessed more than one copy. High rates of infection were necessary to enable sorting of GFP-positive cells by FACS 48-hours post-infection and subsequent plating for limiting dilution analysis. For factor-independent transformation assays, Ba/F3 cells were washed three times and re-suspended in RPMI 1640 with 10% FBS, L-glutamine, fungizone and penicillin-streptomycin. Viable cell counts were obtained using a propidium iodide exclusion on a Guava Personal Cell Analysis System (Millipore). Genomic DNA was harvested using the DNeasy Blood and Tissue Kit (Qiagen).

### Transgene amplification and sequencing

Transgenic DNA was amplified from genomic DNA extracts using vector specific primers (MigFwd - CCCTTTGTACACCCTAAGCCTCCGCC, MigRev - GGAAAGACCCCTAGAATGCTCGTCAA), AccuPrime Taq DNA polymerase, high fidelity (ThermoFisher Scientific) and a modified “slowdown PCR” thermocycler protocol [50]. Sanger sequencing (Eurofins) was performed with transgene-specific internal primers and analyzed using LaserGene 14 Seqman Pro (DNASTAR).

### Limiting dilution analysis

Cells were counted and resuspended in IL-3 free media prior to dilution. Diluted cells were plated in 96-well plates with IL-3-free or IL-3-containing media. Wells were visually inspected once a week for three weeks to identify cell growth. All data was analyzed using the elda function provided with the statmod package (version 1.4.26) in R (version 3.3.2) [38].

### 6-thioguanine survival assay

Ba/F3 cells were cultured in HAT-supplemented media (100µM sodium hypoxanthine, 0.4µM aminopterin, 16µM thymidine, Gibco) for 4 days to select for HPRT-expressing cells. Cells were then allowed to recover for 5 days in HT-supplemented media (100µM sodium hypoxanthine, 16µM thymidine, Gibco) prior to culturing in regular Ba/F3 media. Ba/F3 cells were infected with retrovirus 14 days prior to 6-TG exposure. Positive control cells were treated overnight with 50µg/ml ENU 6 days prior to 6-TG exposure. Biologically replicate lines were screened for 6-TG survival in the following conditions: 2 96-well plates seeded at 1 cell per well without 6-TG (plating efficiency calculations), 2 96-well plates at 1000 cells per well with 20µM 6-TG, and 2 96-well plates at 5000 cells per well with 20µM 6-TG. Wells were visually assessed for growth 14 days later and the number of cells surviving through HPRT-inactivation was determined using ELDA.

## SUPPLEMENTARY FIGURE AND TABLES



## Abbreviations

CSF2RB: Colony stimulating factor 2 receptor beta common subunit
CSF3R: Colony stimulating factor 3 receptor
IL7R: Interleukin 7 receptor
BCR-ABL: Breakpoint cluster region - Abelson tyrosine-protein kinase 1 fusion
CML: Chronic myelogenous leukemia
IL-3: Interleukin-3
FACS: Fluorescence activated cell sorting
ALL: Acute lymphoblastic leukemia
T-ALL: T-cell acute lymphoblastic leukemia
B-ALL: B-cell acute lymphoblastic leukemia
CNL: Chronic neutrophilic leukemia
aCML: Atypical chronic myelogenous leukemia
GFP: Green fluorescent protein
HPRT: Hypoxanthine phosphoribosyltransferase 1
6-TG: 6-thioguanine
ENU: N-ethyl-N-nitrosourea
ELDA: Extreme limiting dilution analysis
Δβ_c_: truncated beta-common receptor (CSF2RB)

## References

[1] Ben-Neriah Y, Daley GQ, Mes-Masson AM, Witte ON, Baltimore D (1986). The chronic myelogenous leukemia-specific P210 protein is the product of the bcr/abl hybrid gene. Science (New York, NY).

[2] Daley GQ, Baltimore D Proceedings of the National Academy of Sciences of the United States of America.

[3] Palacios R, Steinmetz M (1985). Il-3-dependent mouse clones that express B-220 surface antigen, contain Ig genes in germ-line configuration, and generate B lymphocytes in vivo. Cell.

[4] Daley GQ, McLaughlin J, Witte ON, Baltimore D (1987). The CML-specific P210 bcr/abl protein, unlike v-abl, does not transform NIH/3T3 fibroblasts. Science (New York, NY).

[5] Warmuth M, Kim S, Gu XJ, Xia G, Adrian F (2007). Ba/F3 cells and their use in kinase drug discovery. Current opinion in oncology.

[6] Yuzugullu H, Thorpe LM, Von T, Walker SR, Roberts TM, Frank DA, Zhao JJ (2016). NTRK2 activation cooperates with PTEN deficiency in T-ALL through activation of both the PI3K-AKT and JAK-STAT3 pathways. Cell discovery.

[7] Cante-Barrett K, Spijkers-Hagelstein JA, Buijs-Gladdines JG, Uitdehaag JC, Smits WK, van der Zwet J, Buijsman RC, Zaman GJ, Pieters R, Meijerink JP (2016). MEK and PI3K-AKT inhibitors synergistically block activated IL7 receptor signaling in T-cell acute lymphoblastic leukemia. Leukemia.

[8] White Y, Bagchi A, Van Ziffle J, Inguva A, Bollag G, Zhang C, Carias H, Dickens D, Loh M, Shannon K, Firestone AJ (2016). KRAS insertion mutations are oncogenic and exhibit distinct functional properties. Nature communications.

[9] Ishibashi T, Yaguchi A, Terada K, Ueno-Yokohata H, Tomita O, Iijima K, Kobayashi K, Okita H, Fujimura J, Ohki K, Shimizu T, Kiyokawa N (2016). Ph-like ALL-related novel fusion kinase ATF7IP-PDGFRB exhibits high sensitivity to tyrosine kinase inhibitors in murine cells. Experimental hematology.

[10] Yang S, Luo C, Gu Q, Xu Q, Wang G, Sun H, Qian Z, Tan Y, Qin Y, Shen Y, Xu X, Chen SH, Chan CC, Wang H, Mao M, Fang DD (2016). Activating JAK1 mutation may predict the sensitivity of JAK-STAT inhibition in hepatocellular carcinoma. Oncotarget.

[11] Reshetnyak AV, Murray PB, Shi X, Mo ES, Mohanty J, Tome F, Bai H, Gunel M, Lax I, Schlessinger J Proceedings of the National Academy of Sciences of the United States of America..

[12] Kiessling MK, Curioni-Fontecedro A, Samaras P, Atrott K, Cosin-Roger J, Lang S, Scharl M, Rogler G (2015). Mutant HRAS as novel target for MEK and mTOR inhibitors. Oncotarget.

[13] Arts FA, Chand D, Pecquet C, Velghe AI, Constantinescu S, Hallberg B, Demoulin JB (2016). PDGFRB mutants found in patients with familial infantile myofibromatosis or overgrowth syndrome are oncogenic and sensitive to imatinib. Oncogene.

[14] Cheng H, Zou Y, Ross JS, Wang K, Liu X, Halmos B, Ali SM, Liu H, Verma A, Montagna C, Chachoua A, Goel S, Schwartz EL, Zhu C, Shan J, Yu Y (2015). RICTOR Amplification Defines a Novel Subset of Patients with Lung Cancer Who May Benefit from Treatment with mTORC1/2 Inhibitors. Cancer discovery.

[15] Roncero AM, Lopez-Nieva P, Cobos-Fernandez MA, Villa-Morales M, Gonzalez-Sanchez L, Lopez-Lorenzo JL, Llamas P, Ayuso C, Rodriguez-Pinilla SM, Arriba MC, Piris MA, Fernandez-Navarro P, Fernandez AF, Fraga MF, Santos J, Fernandez-Piqueras J (2016). Contribution of JAK2 mutations to T-cell lymphoblastic lymphoma development. Leukemia.

[16] Kobayashi Y, Togashi Y, Yatabe Y, Mizuuchi H, Jangchul P, Kondo C, Shimoji M, Sato K, Suda K, Tomizawa K, Takemoto T, Hida T, Nishio K, Mitsudomi T (2015). EGFR Exon 18 Mutations in Lung Cancer: Molecular Predictors of Augmented Sensitivity to Afatinib or Neratinib as Compared with First- or Third-Generation TKIs. Clinical cancer research.

[17] Lindblad O, Kazi JU, Ronnstrand L, Sun J (2015). PI3 kinase is indispensable for oncogenic transformation by the V560D mutant of c-Kit in a kinase-independent manner. Cellular and molecular life sciences.

[18] Grubbs EG, Ng PK, Bui J, Busaidy NL, Chen K, Lee JE, Lu X, Lu H, Meric-Bernstam F, Mills GB, Palmer G, Perrier ND, Scott KL, Shaw KR, Waguespack SG, Williams MD (2015). RET fusion as a novel driver of medullary thyroid carcinoma. The Journal of clinical endocrinology and metabolism.

[19] Stockklausner C, Klotter AC, Dickemann N, Kuhlee IN, Duffert CM, Kerber C, Gehring NH, Kulozik AE (2015). The thrombopoietin receptor P106L mutation functionally separates receptor signaling activity from thrombopoietin homeostasis. Blood.

[20] Schinnerl D, Fortschegger K, Kauer M, Marchante JR, Kofler R, Den Boer ML, Strehl S (2015). The role of the Janus-faced transcription factor PAX5-JAK2 in acute lymphoblastic leukemia. Blood.

[21] Capelletti M, Dodge ME, Ercan D, Hammerman PS, Park SI, Kim J, Sasaki H, Jablons DM, Lipson D, Young L, Stephens PJ, Miller VA, Lindeman NI, Munir KJ, Richards WG, Janne PA (2014). Identification of recurrent FGFR3-TACC3 fusion oncogenes from lung adenocarcinoma. Clinical cancer research.

[22] Degryse S, de Bock CE, Cox L, Demeyer S, Gielen O, Mentens N, Jacobs K, Geerdens E, Gianfelici V, Hulselmans G, Fiers M, Aerts S, Meijerink JP, Tousseyn T, Cools J (2014). JAK3 mutants transform hematopoietic cells through JAK1 activation, causing T-cell acute lymphoblastic leukemia in a mouse model. Blood.

[23] Yin C, Sandoval C, Baeg GH (2015). Identification of mutant alleles of JAK3 in pediatric patients with acute lymphoblastic leukemia. Leukemia & lymphoma.

[24] Kiel MJ, Velusamy T, Rolland D, Sahasrabuddhe AA, Chung F, Bailey NG, Schrader A, Li B, Li JZ, Ozel AB, Betz BL, Miranda RN, Medeiros LJ, Zhao L, Herling M, Lim MS (2014). Integrated genomic sequencing reveals mutational landscape of T-cell prolymphocytic leukemia. Blood.

[25] Janke H, Pastore F, Schumacher D, Herold T, Hopfner KP, Schneider S, Berdel WE, Buchner T, Woermann BJ, Subklewe M, Bohlander SK, Hiddemann W, Spiekermann K, Polzer H (2014). Activating FLT3 mutants show distinct gain-of-function phenotypes in vitro and a characteristic signaling pathway profile associated with prognosis in acute myeloid leukemia. PloS one.

[26] Marty C, Saint-Martin C, Pecquet C, Grosjean S, Saliba J, Mouton C, Leroy E, Harutyunyan AS, Abgrall JF, Favier R, Toussaint A, Solary E, Kralovics R, Constantinescu SN, Najman A, Vainchenker W (2014). Germ-line JAK2 mutations in the kinase domain are responsible for hereditary thrombocytosis and are resistant to JAK2 and HSP90 inhibitors. Blood.

[27] Tomita O, Iijima K, Ishibashi T, Osumi T, Kobayashi K, Okita H, Saito M, Mori T, Shimizu T, Kiyokawa N (2014). Sensitivity of SNX2-ABL1 toward tyrosine kinase inhibitors distinct from that of BCR-ABL1. Leukemia research.

[28] Bossi D, Carlomagno F, Pallavicini I, Pruneri G, Trubia M, Raviele PR, Marinelli A, Anaganti S, Cox MC, Viale G, Santoro M, Di Fiore PP, Minucci S (2014). Functional characterization of a novel FGFR1OP-RET rearrangement in hematopoietic malignancies. Molecular oncology.

[29] Velghe AI, Van Cauwenberghe S, Polyansky AA, Chand D, Montano-Almendras CP, Charni S, Hallberg B, Essaghir A, Demoulin JB (2014). PDGFRA alterations in cancer: characterization of a gain-of-function V536E transmembrane mutant as well as loss-of-function and passenger mutations. Oncogene.

[30] Watanabe-Smith K, Tognon C, Tyner JW, Meijerink JP, Druker BJ, Agarwal A (2016). Discovery and functional characterization of a germline, CSF2RB-activating mutation in leukemia. Leukemia.

[31] Jenkins BJ, D'Andrea R, Gonda TJ (1995). Activating point mutations in the common beta subunit of the human GM-CSF, IL-3 and IL-5 receptors suggest the involvement of beta subunit dimerization and cell type-specific molecules in signalling. The EMBO journal.

[32] Prassolov V, Meyer J, Brandenburg G, Hannemann J, Bergemann J, Ostertag W, Stocking C (2001). Functional identification of secondary mutations inducing autonomous growth in synergy with a truncated interleukin-3 receptor: implications for multi-step oncogenesis. Experimental hematology.

[33] Maxson JE, Gotlib J, Pollyea DA, Fleischman AG, Agarwal A, Eide CA, Bottomly D, Wilmot B, McWeeney SK, Tognon CE, Pond JB, Collins RH, Goueli B, Oh ST, Deininger MW, Chang BH (2013). Oncogenic CSF3R mutations in chronic neutrophilic leukemia and atypical CML. The New England journal of medicine.

[34] Dong F, Brynes RK, Tidow N, Welte K, Lowenberg B, Touw IP (1995). Mutations in the gene for the granulocyte colony-stimulating-factor receptor in patients with acute myeloid leukemia preceded by severe congenital neutropenia. The New England journal of medicine.

[35] Gits J, van Leeuwen D, Carroll HP, Touw IP, Ward AC (2006). Multiple pathways contribute to the hyperproliferative responses from truncated granulocyte colony-stimulating factor receptors. Leukemia.

[36] Shochat C, Tal N, Bandapalli OR, Palmi C, Ganmore I, te Kronnie G, Cario G, Cazzaniga G, Kulozik AE, Stanulla M, Schrappe M, Biondi A, Basso G, Bercovich D, Muckenthaler MU, Izraeli S (2011). Gain-of-function mutations in interleukin-7 receptor-α (IL7R) in childhood acute lymphoblastic leukemias. The Journal of Experimental Medicine.

[37] Zenatti PP, Ribeiro D, Li W, Zuurbier L, Silva MC, Paganin M, Tritapoe J, Hixon JA, Silveira AB, Cardoso BA, Sarmento LM, Correia N, Toribio ML, Kobarg J, Horstmann M, Pieters R (2011). Oncogenic IL7R gain-of-function mutations in childhood T-cell acute lymphoblastic leukemia. Nature genetics.

[38] Hu Y, Smyth GK (2009). ELDA: extreme limiting dilution analysis for comparing depleted and enriched populations in stem cell and other assays. Journal of immunological methods.

[39] Jenkins BJ, Blake TJ, Gonda TJ (1998). Saturation mutagenesis of the beta subunit of the human granulocyte-macrophage colony-stimulating factor receptor shows clustering of constitutive mutations, activation of ERK MAP kinase and STAT pathways, and differential beta subunit tyrosine phosphorylation. Blood.

[40] Kazi JU, Sun J, Ronnstrand L (2013). The presence or absence of IL-3 during long-term culture of Flt3-ITD and c-Kit-D816V expressing Ba/F3 cells influences signaling outcome. Experimental hematology.

[41] Drake JW Rates of spontaneous mutation among RNA viruses. Proceedings of the National Academy of Sciences of the United States of America.

[42] Davidson CJ, Zeringer E, Champion KJ, Gauthier MP, Wang F, Boonyaratanakornkit J, Jones JR, Schreiber E (2012). Improving the limit of detection for Sanger sequencing: A comparison of methodologies for KRAS variant detection. BioTechniques.

[43] Hannemann J, Hara T, Kawai M, Miyajima A, Ostertag W, Stocking C (1995). Sequential mutations in the interleukin-3 (IL3)/granulocyte-macrophage colony-stimulating factor/IL5 receptor beta-subunit genes are necessary for the complete conversion to growth autonomy mediated by a truncated beta C subunit. Molecular and cellular biology.

[44] Hornakova T, Springuel L, Devreux J, Dusa A, Constantinescu SN, Knoops L, Renauld JC (2011). Oncogenic JAK1 and JAK2-activating mutations resistant to ATP-competitive inhibitors. Haematologica.

[45] Pikman Y, Lee BH, Mercher T, McDowell E, Ebert BL, Gozo M, Cuker A, Wernig G, Moore S, Galinsky I, DeAngelo DJ, Clark JJ, Lee SJ, Golub TR, Wadleigh M, Gilliland DG (2006). MPLW515L is a novel somatic activating mutation in myelofibrosis with myeloid metaplasia. PLoS medicine.

[46] Hirota S, Isozaki K, Moriyama Y, Hashimoto K, Nishida T, Ishiguro S, Kawano K, Hanada M, Kurata A, Takeda M, Muhammad Tunio G, Matsuzawa Y, Kanakura Y, Shinomura Y, Kitamura Y (1998). Gain-of-function mutations of c-kit in human gastrointestinal stromal tumors. Science (New York, NY).

[47] Greulich H, Kaplan B, Mertins P, Chen TH, Tanaka KE, Yun CH, Zhang X, Lee SH, Cho J, Ambrogio L, Liao R, Imielinski M, Banerji S, Berger AH, Lawrence MS, Zhang J Proceedings of the National Academy of Sciences of the United States of America..

[48] Stocking C, Loliger C, Kawai M, Suciu S, Gough N, Ostertag W (1988). Identification of genes involved in growth autonomy of hematopoietic cells by analysis of factor-independent mutants. Cell.

[49] Hawley RG, Lieu FH, Fong AZ, Hawley TS (1994). Versatile retroviral vectors for potential use in gene therapy. Gene therapy.

[50] Frey UH, Bachmann HS, Peters J, Siffert W (2008). PCR-amplification of GC-rich regions: ‘slowdown PCR’. Nature protocols.

