# Supplementary material for: Analysis of acquired mutations in transgenes arising in Ba/F3 transformation assays: findings and recommendations
Source: Oncotarget. 2017 Feb 16;8(8):12596–606. doi: 10.18632/oncotarget.15392 (PMC5355038; doi:10.18632/oncotarget.15392)
Supplement: Supplementary file 1 [file oncotarget-08-12596-s001.pdf]

Analysis of acquired mutations in transgenes arising in Ba/F3 transformation assays: findings and recommendations

Supplementary Material

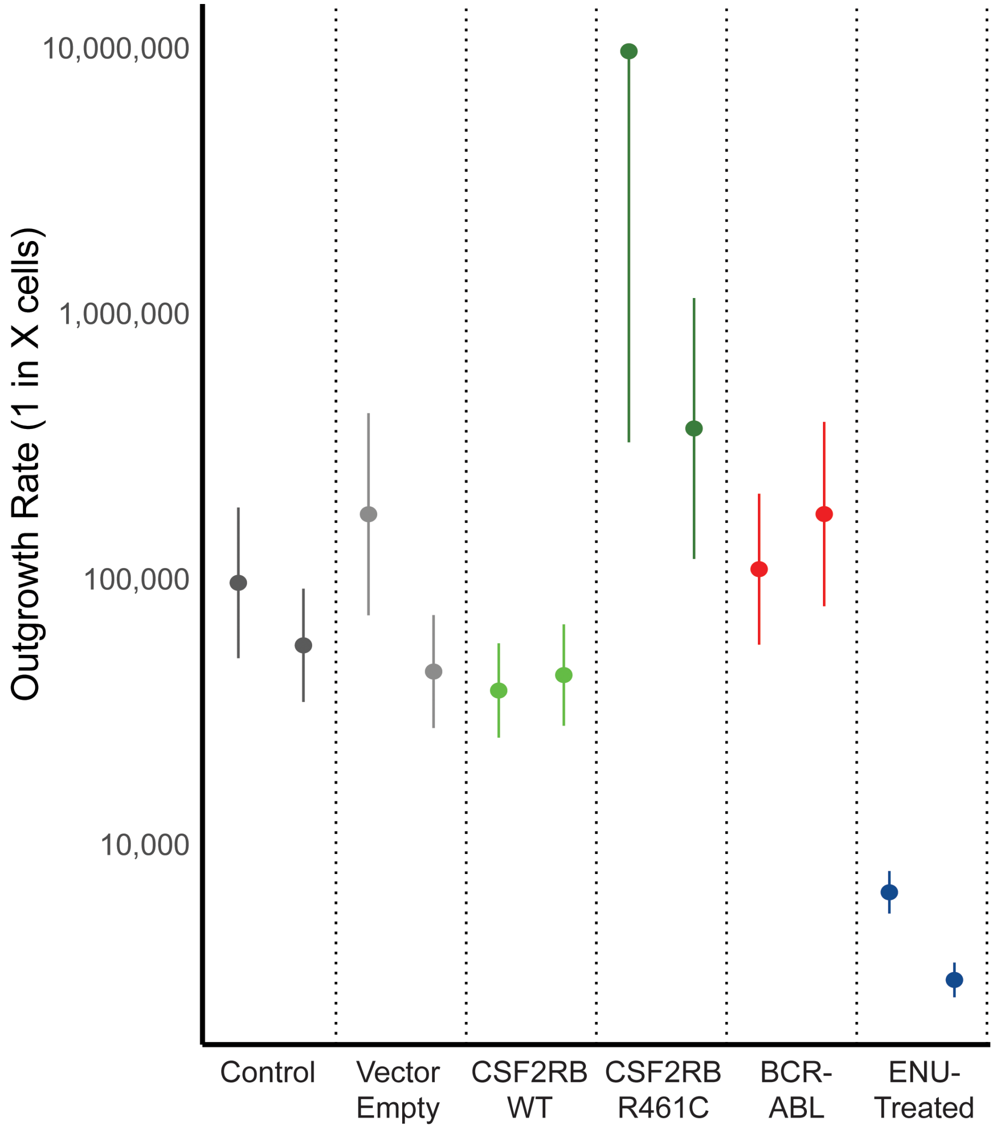

Supplementary Figure 1: Ba/F3 cells expressing CSF2RB R461C do not demonstrate increased rates of mutagenesis. Outgrowth rate and 95% confidence intervals are shown for biologically replicate lines. Outgrowth rate is expressed as 1 in X cells have mutationally inactivated HPRT to survive 6-TG treatment, thus a higher outgrowth rate indicates a less mutagenic condition. Mutagenesis induced by ENU-treatment was used as a positive control.

Supplementary Table 1: Studies using the Ba/F3 transformation assay (2014-2016) – Frequency of sequencing outgrown Ba/F3 lines

PMID	Year	Journal	Title	Method of transduction	Sequenced transgene of outgrown lines
27672444	2016	Cell Discov	NTRK2 activation cooperates with PTEN deficiency in T-ALL through activation of both the PI3K-AKT and JAK-STAT3 pathways.	Retrovirus	No
27174491	2016	Leukemia	MEK and PI3K-AKT inhibitors synergistically block activated IL7 receptor signaling in T-cell acute lymphoblastic leukemia.	Electroporation	No
26854029	2016	Nat Commun	KRAS insertion mutations are oncogenic and exhibit distinct functional properties.	Retrovirus	No
26703895	2016	Exp Hematol	Ph-like ALL-related novel fusion kinase ATF7IP-PDGFRB exhibits high sensitivity to tyrosine kinase inhibitors in murine cells.	Retrovirus	No
26701727	2016	Oncotarget	Activating JAK1 mutation may predict the sensitivity of JAK-STAT inhibition in hepatocellular carcinoma.	Lentivirus	No
26630010	2015	PNAS	Augmentor α and β (FAM150) are ligands of the receptor tyrosine kinases ALK and LTK: Hierarchy and specificity of ligand-receptor interactions.	Retrovirus	No
26544513	2015	Oncotarget	Mutant HRAS as novel target for MEK and mTOR inhibitors.	Retrovirus	No
26455322	2016	Oncogene	PDGFRB mutants found in patients with familial infantile myofibromatosis or overgrowth syndrome are oncogenic and sensitive to imatinib.	Electroporation	No
26370156	2015	Cancer Discov	RICTOR Amplification Defines a Novel Subset of Patients with Lung Cancer Who May Benefit from Treatment with mTORC1/2 Inhibitors.	Unclear	No
26216197	2016	Leukemia	Contribution of JAK2 mutations to T-cell lymphoblastic lymphoma development.	Lentivirus	No
26206867	2015	Clin Cancer Res	EGFR Exon 18 Mutations in Lung Cancer: Molecular Predictors of Augmented Sensitivity to Afatinib or Neratinib as Compared with First- or Third-Generation TKIs.	Retrovirus	No
26040420	2015	Cell Mol Life Sci	PI3 kinase is indispensable for oncogenic transformation by the V560D mutant of c-Kit in a kinase-independent manner.	Retrovirus	No
25546157	2015	J Clin Endocrinol Metab	RET fusion as a novel driver of medullary thyroid carcinoma.	Unclear	No
25538044	2015	Blood	The thrombopoietin receptor P106L mutation functionally separates receptor signaling activity from thrombopoietin homeostasis.	Electroporation	No
25515960	2015	Blood	The role of the Janus-faced transcription factor PAX5-JAK2 in acute lymphoblastic leukemia.	Electroporation	No
25294908	2014	Clin Cancer Res	Identification of recurrent FGFR3-TACC3 fusion oncogenes from lung adenocarcinoma.	Retrovirus	No
25193870	2014	Blood	JAK3 mutants transform hematopoietic cells through JAK1 activation, causing T-cell acute lymphoblastic leukemia in a mouse model.	Retrovirus	No
25146434	2015	Leuk Lymphoma	Identification of mutant alleles of JAK3 in pediatric patients with acute lymphoblastic leukemia.	Electroporation	No
24825865	2014	Blood	Integrated genomic sequencing reveals mutational landscape of T-cell prolymphocytic leukemia.	Unclear	No
24608088	2014	PLoS One	Activating FLT3 mutants show distinct gain-of-function phenotypes in vitro and a characteristic signaling pathway profile associated with prognosis in acute myeloid leukemia.	Unclear	No
24398328	2014	Blood	Germ-line JAK2 mutations in the kinase domain are responsible for hereditary thrombocytosis and are resistant to JAK2 and HSP90 inhibitors.	Retrovirus	No
24367893	2014	Leuk Res	Sensitivity of SNX2-ABL1 toward tyrosine kinase inhibitors distinct from that of BCR-ABL1.	Retrovirus	No
24315414	2014	Mol Oncol	Functional characterization of a novel FGFR1OP-RET rearrangement in hematopoietic malignancies.	Retrovirus	No
23752188	2014	Oncogene	PDGFRA alterations in cancer: characterization of a gain-of-function V536E transmembrane mutant as well as loss-of-function and passenger mutations.	Electroporation	No

Source: PubMed articles matching "Ba/F3" or "Baf3", published 2014-2016, and using Ba/F3 cells for the purpose to establish transformative potential of genetic products. Data current as of 10-26-16

Supplementary Table 2: Sequence results of outgrown Ba/F3 lines from bulk withdrawal assays. Where a mixed read is evident (double peaks on Sanger trace) the estimated mutational burden is provided. This includes one cell line where Sanger sequencing detected a near-heterozygous deletion, alongside a point mutation within the deleted region on the non-deleted copy.

Gene	Variant	Il-3 independent transformation	Baseline gDNA	Cultured gDNA	Withdrawn gDNA #1	Withdrawn gDNA #2
CSF2RB	WT	+	✓	n/a	V449E	V449E
		-				
		-				
	R461C	+	✓	n/a	Del H43-S417	Del H43-S417
		+	✓	n/a	K389R, YN420CS, R657G	K389R, YN420CS, R657G
		+	✓	n/a	Del S161-S438	100% - Del L30-M81<br>50% - T27T, P118T, G285R, L626L, E653K
		+	✓	✓	50% - F451S	50% - F451S
		+	✓	✓	✓	✓
CSF3R	WT	+	✓	n/a	S581C	S581C
		+	✓	✓	80% - E524K	80% - E524K
		+	✓	✓	50% - Del A33-P558	50% - Del A33-P558
		+	✓	✓	50% - T618I	50% - T618I
	T618I	+	✓	n/a	✓	✓
		+	✓	✓	✓	✓
		+	✓	✓	✓	✓
		+	✓	✓	✓	✓
	W791X	+	✓	n/a	Internal 111aa duplication (bounds unclear)	Internal 111aa duplication (bounds unclear)
		+	✓	✓	50% - E524K,<br>50% - Del L38-T609	50% - E524K,<br>50% - Del L38-T609
		+	✓	✓	✓	✓
		+	✓	50% - Del W187-I564	50% - Del L129-E622	50% - Del L129-E622
IL7R	WT	-				
		-				
		-				
		-				
	Ins243PPCL	+	✓	n/a	✓	✓
		+	✓	✓	✓	✓
		+	✓	✓	✓	✓

		+	✓	✓	✓		✓
Vector	Empty	+	✓	✓	Contamination: Ins243PPCL	IL7R	Contamination: IL7R Ins243PPCL
		-					
		-					
		-					

✓ - Full transgene sequenced, no variants detected
